# Ethical problems in nursing management – a cross-sectional survey about solving problems

**DOI:** 10.1186/s12913-019-4245-4

**Published:** 2019-06-24

**Authors:** Elina Aitamaa, Riitta Suhonen, Pauli Puukka, Helena Leino-Kilpi

**Affiliations:** 1 Department of Nursing Science, University of Turku, FI-20014 Turku, Finland; 2 Welfare Division, Turku University Hospital and City of Turku, Turku, Finland; 3 The National Institute for Health and Welfare, Turku, Finland; 4 University of Turku, Turku, Finland; 5 Turku University Hospital, Turku, Finland

**Keywords:** Ethical problem, Solving method, Nursing management, Survey

## Abstract

**Background:**

Nurse managers encounter a wide range of ethical problems related to patients, staff, the organisation and themselves. However, little is known about the methods they use to try to solve these problems. In this study, our goal is to fill this knowledge gap by investigating the ethical problems encountered by nurse managers, the frequency of use and usefulness of different methods to solve these problems, and the background factors associated with the use of the methods.

**Methods:**

A cross-sectional survey study was conducted in November 2014–May 2015 in Finland. The data were collected from nurse managers in strategic, middle and ward management (*n* = 214) using a questionnaire developed for this study. The questionnaire consisted of four parts: socio-demographic background factors, frequency and difficulty of ethical problems in nursing management, frequency of use and usefulness of the methods in solving ethical problems, and work-related background factors.

**Results:**

Discussions with nurses was the most frequently used method, used by 94% of the nurse managers either often or always in the case of ethical problems, followed by the use of personal values (74%) and discussions with manager colleagues (70%). However, almost all methods in the different groups – discussion and deliberation, use of outside experts, written instructions and ethical principles, acts and degrees as well as work arrangements – were considered somewhat or very useful by more than half of the respondents. The use of outside experts was the least used and the least useful method.

**Conclusions:**

When solving ethical problems, nurse managers use most frequently the same methods as a few decades ago. A more diverse range of methods would be helpful in ethical problem-solving. The use of outside experts, ethics literature and codes of ethics should be combined with ethical reasoning and decision-making to get new dimensions and outside knowledge.

## Background

Nurse managers (NMs) encounter various ethical problems in their work in connection with both their managerial and nursing care duties [1]. The problems are related to patients, staff, the organisation and the NMs themselves [2]. Ethical problems are encountered at least weekly by about half [1] or more than half [3] of NMs. Ethical problems and moral distress are found to be associated with adverse outcomes in organisations [4, 5]. For these reasons, this research topic is important.

There are ethical decision-making models designed to support analysing and solving ethical problems. Thompson et al. (2006) recommend the DECIDE model consisting of six steps: 1. Define problems – What is the ethical issue?, 2. Ethical review – What principles are relevant to the case?, 3. Consider options – What can be done?, 4. Investigate – Ethical outcomes, costs and benefits, 5. Decide on an action – plan and objectives, 6. Evaluate results – against objectives [6]. To answer these questions and to find alternatives and appropriate actions, other methods such as consultations, literature and laws are required.

Studies about the activities, aids and resources used by NMs for solving ethical problems have been conducted since the 1990s, but in many studies, the pre-identified methods were at quite a general level and the frequency of use is not well known. NMs have identified some activities they use to solve work-related ethical problems: using discussion, cooperation, the work organisation, intervention, personal values, an operational model, statistics and feedback, and personal examples. Discussion and cooperation were the most commonly used activities [7]. When solving ethical problems, NMs’ own personal values have been found to be the most useful aid [8–10], or at least among the three most useful ones [7, 10]. Discussions are also among the most frequently used resources in ethical problem-solving [7, 10, 11]. NMs discuss ethical problems most often with nursing colleagues and administrative colleagues [10, 11], but also with patient’s physician [1, 11] and other professionals [11] or different parties involved in the problematic issue [7].

Ethics committees, written guidelines and codes are also used in ethical problem-solving, but less frequently than own values and discussions [1, 8, 9]. Ethics committees or ethics consultants are only available in some healthcare institutions [12]. Not all staff members in healthcare organisations are aware of the existence of the ethics committee in the institution, and those who know about it have different views of its role [13]. Act on the rights of patients, the organisation’s own statements on ethics as well as ethics literature are mentioned as potential aids when dealing with ethical problems [8–10].

Professional codes of ethics are often mentioned as a guide for ethical issues in nursing. These codes have also been tailored for the needs of nurse managers, e.g. The Proto-code of Ethics and Conduct for European Nurse Directors [14] in Europe and The Code of Ethics for Nurse Managers in Finland [15]. The use of these codes has only been investigated to a limited extent. In Finland, an earlier study [3] found that professional codes of ethics are most frequently used with ethical problems concerning patient care. NMs in middle or strategic management use codes of ethics more often than nurse managers in charge of a ward. NMs also use codes of ethics intended for nurses or other health care professions more frequently than managers’ own codes [3].

Recently, moral case deliberation (MCD) and ethics rounds have been reported as methods consisting of guided and structured discussions concerning moral cases, moral dilemmas and questions of good care [16, 17]. Studies about the use of these methods are few and they mainly deal with health care professionals and ethical issues in patient care. Moral case deliberations have been evaluated positively [17]. Ethics rounds are considered to provide new perspectives and insights into ethical issues, but not to affect daily work [16].

The usefulness of the methods is poorly known. Discussions and personal values are often used, but there seem to be no studies about the usefulness of them. Codes of ethics are criticised for being too restrictive, but also too vague to be of any real help in practical situations [18]. However, it is extremely important to determine ethical problems in NMs’ work and to know what kind of methods are the most useful to develop NMs’ ethical decision-making and to implement a systematic model into practice.

The aims of this study were to identify the most often used and the most useful methods for solving ethical problems in NMs’ work and to determine the background factors associated with the frequency of use or usefulness of the methods. In addition, the aim was to determine the categories of ethical problems in NMs’ work which are associated with the frequency of use or usefulness of the methods.

## Methods

Cross-sectional survey design was used in this descriptive and comparative nationwide study. The target population consists of all nurse managers in public healthcare in Finland. The study was carried out from November 2014 to May 2015.

Participants (*n* = 214) represented ward, middle and strategic management. They were selected step by step to obtain a total of 200 respondents based on the power analysis. The sample size calculations were based on expected group differences and the results (means and standard deviations) of a previous study [3]. With group difference of 10%, significance level of 0.05 and power 0.80, the estimated sample size was 200.

First, the health care organizations were randomly selected one at a time. From primary health care, four organisations in the 10 largest cities according to the number of inhabitants were selected, and from specialised health care, one university hospital district out of five and four hospital districts out of 20 were selected. In five health care organisations, the target group included all NMs in ward management, and in four organisations, all nurse managers at all management levels. In selected organizations, all NMs were included. Members of the national association of academic nurse managers and experts and participants of one education meeting targeted at nurse managers were also included in the study.

The questionnaire Ethical Problems in Nursing Management (EProNuMa) was developed for this study as no suitable instrument was available. The items of the instrument are based on literature review and interviews. One part of the EProNuMa was adopted from a previously used instrument of Ikola-Norrbacka [19] in order to find out if they have associations with ethical problems. The content validity of the EProNuMa was assessed by 5 NMs with different types of work experience at different levels of nursing management. A pilot test was conducted by 15 nurse managers.

The EProNuMa included four parts. The first one consisted of the respondents’ socio-demographic background factors (gender, age, position in organisation, experience in health care management, number of subordinates in nursing positions*).* The second part consisted of questions related to ethical problems (list of 65 response options) (see [20]). The third part consisted of questions about methods used for solving ethical problems. NMs were asked how often they used the method (never, very rarely, sometimes, often, always, cannot say) and how useful they considered the method (very useless, somewhat useless, somewhat useful, very useful, cannot say). The methods were divided into five groups: discussion and deliberation (12 items, Cronbach’s α: frequency 0.77, usefulness 0.81), use of outside experts (7 items, Cronbach’s α: frequency 0.71, usefulness 0.85), written instructions and ethical principles (8 items. Cronbach’s α: frequency 0.87, usefulness 0.89), acts and degrees (5 items, Cronbach’s α: frequency 0.83, usefulness 0.90) and work arrangements (8 items, Cronbach’s α: frequency 0.73, usefulness 0.82). The fourth part of the instrument included 9 statements of work-related background factors with the scale “totally disagree” – “totally agree” with a neutral midpoint. The statements were from the original questionnaire of Ikola-Norrbacka [19].

A link to the electronic survey was sent by e-mail to 855 NMs and a paper survey to 231 NMs. Two reminders were sent by email to all respondents. Of the total of 1086 questionnaires, 214 were returned, giving a response rate of 20%. Data collection was carried out so that the researcher did not know from whom or from what organisation the responses came.

### Ethical considerations

Ethical approval for the study was given by the Ethics Committee of the University (22/2014) and permissions to collect data were given according to national standards at all participating organisations [21]. An information letter about the study was also sent to the respondents. The respondents were informed about anonymous response and confidentiality. Voluntary participation and the aim of the study were explained to them. Permission to use parts of a previously developed instrument was given by the original developer Ikola-Norrbacka [19].

### Data analysis

Data were analysed using SAS 9.3 statistical software package (SAS Institute Inc., Cary, North Carolina, USA). Descriptive statistics, such as frequencies, means, medians and standard deviations, were used to describe the variables. In the sum score of work-related background factors, the scales of two negative statements were reversed before combining to the sum score. The internal consistency of the sum variables was examined by Cronbach’s alpha coefficient. The differences between the means of the sum variables were analysed by repeated measures analysis of variance. The Tukey method was used to adjust for multiple comparisons. Multivariable linear regression analyses were used to find out which independent factors were associated with the frequency of use of the methods and the usefulness of the methods in solving ethical problems. A separate linear regression model was formed for each method. At the beginning of the analyses, all background factors and the frequency and difficulty of all ethical problems encountered were used as independent variables in the models. Then, using backward variable selection method the non-significant predictors were removed. *P*-values less than 0.05 were considered statistically significant.

## Results

### Ethical problems encountered by NMs

The frequency and difficulty of ethical problems in NMs’ work were also investigated in this study and reported in detail in a separate article [20]. Sum scores were reported of groups of ethical problems related to patients (mean: frequency 2.16, difficulty 3.26), nursing staff (mean: frequency 2.53, difficulty 3.12), other professional groups (mean: frequency 2.14, difficulty 3.59), the organisation (mean: frequency 2.52, difficulty 3.92) and the NMs themselves (mean: frequency 2.08, difficulty 3.44). The most often encountered problems were related to nursing staff and the organisation. The organisation-related ethical problems were most difficult [20]. In this study, the groups of ethical problems are considered as background factors.

### Participants

The respondents were NMs working at different levels of management: in charge of a ward (75%), middle (19%) or strategic management (6%). The majority of respondents were female (92%) and their age ranged from 29 to 66 years (mean 52). The number of their nurse subordinates in nursing position ranged from 3 to 1100 (mean 79, median 30). At middle or strategic management, NMs had on average 230 subordinates while NMs in charge of a ward had on average 31 subordinates. Respondents’ average experience in health care management was 12 years (range 0.3–34).

### Work-related background factors

Work-related background factors consisted of statements concerning NMs’ working conditions and the organisation from an ethical point of view. Most NMs agreed that their work is compatible with their values (86%) and they see their work as meaningful (85%). Instead, only 37% agreed that the values of different professions in their organisation are compatible. The majority of NMs (79%) also agreed that they had enough decision-making power in their work. More than half of the NMs agreed that accelerating pace of work (69%) and insufficient financial resources (56%) make it more difficult to focus on what is essential in the work.

Two statements were about the management of unethical situations in the organisation. About half of the respondents (52%) agreed that their organisation addresses unethical situations in an appropriate manner. About one third (32%) agreed that their organisation has functioning instructions for dealing with unethical situations. All items of work-related background factors were combined to a sum score (mean 3.30, SD 0.52, range 1.89–4.67).

### Most frequently used methods of solving ethical problems

The highest sum variable score was reported for the frequency of using discussions and deliberation and the lowest for using outside experts when solving ethical problems. The differences between discussions and deliberation and the methods in all the other groups were statistically significant, as were the differences between using outside experts and all other methods. The other methods were acts and degrees, written instructions and principles and work arrangements (Table 1).

**Table 1:** Frequency of use and usefulness of methods of solving ethical problems (sum variable scores)

Sum variables	No.	Mean	Median	SD	Min.	Max.	Cronbach’s α
Frequency of using methods (range 1–5)							
a. Discussion and deliberation (12 items) ^a^	214	3.19	3.09	0.50	1.92	4.67	0.771
b. Use of outside experts (7 items) ^b^	214	2.08	2.00	0.52	1.00	3.43	0.708
c. Written instructions and ethical principles (8 items)	213	2.73	2.67	0.75	1.13	4.50	0.875
d. Acts and degrees (5 items)	211	2.85	2.75	0.89	1.00	5.00	0.829
e. Work arrangements (8 items)	214	2.83	2.88	0.57	1.13	4.25	0.734
Usefulness of methods (range 1–4)							
f. Discussion and deliberation (12 items) ^c^	213	3.56	3.60	0.33	2.56	4.00	0.806
g. Use of outside experts (7 items) ^d^	210	3.12	3.00	0.51	1.57	4.00	0.849
h. Written instructions and ethical principles (8 items)^e^	207	3.30	3.33	0.47	2.00	4.00	0.890
i. Acts and degrees (5 items)	203	3.50	3.60	0.48	2.00	4.00	0.899
j. Work arrangements (8 items)	210	3.41	3.41	0.43	2.00	4.00	0.822

^a^ Mean value higher compared to sum variables b, c, d and e, *p* < .0001

^b^ Mean value lower compared to sum variables a, c, d and e, *p* < .0001

^c^ Mean value higher compared to sum variables g and h, *p* < .0001, and sum variable j, *p* = 0.0004

^d^ Mean value lower compared to sum variables f, h, i and j, *p* < .0001

^e^ Mean value lower compared to sum variable i, *p* < .0001, and sum variable j, *p* = 0.0071

When single methods were examined, discussion with nurses was clearly the most frequently used method, as 94% of the NMs used it often or always when trying to solve ethical problems. Relying on own personal values, discussion with manager colleagues or with own manager were the next most frequently used methods, all of them used often or always by 65% or more of the NMs. The Act on the Status and Rights of Patients (785/1992) was the most often used act, being the 7th most frequently used method. Out of written instructions, the most frequently used were situation-specific, pre-defined instructions and the organisation’s explicitly stated values or ethical principles, which were among the ten most often used methods. Instead, the professional codes of ethics and the code of ethics for nurse managers (often 12%, always 2% were used less often (Table 2).

**Table 2:** Frequency and usefulness of methods of solving ethical problems

Method of solving ethical problem		Frequency of use (scale 1–5)^a^						Usefulness (scale 1–4)^b^						
	categ.	Mean	Median	SD	often %	always %	n	order of usefulness	Mean	Median	SD	somewhat useful	very useful	n
1. Discussion with nurses	D	4.36	4.00	0.63	51	43	213	1.	3.83	4.00	0,37	17	83	211
2. Relying on personal values	D	3.96	4.00	0.93	43	31	209	12.	3.55	4.00	0.58	38	58	200
3. Discussion with manager colleagues	D	3.77	4.00	0.78	56	14	214	4.	3.68	4.00	0.52	29	69	210
4. Discussion with own manager	D	3.74	4.00	0.82	50	15	214	6.	3.62	4.00	0.62	27	68	207
5. Situation-specific, pre-defined instructions	I	3.40	4.00	1.07	38	14	203	13.	3.55	4.00	0.59	36	59	185
6.Discussing with the doctor treating the patient	D	3.34	3.00	1.01	28	11	207	11.	3.55	4.00	0.58	37	59	192
7. Act on the Status and Rights of Patients (785/1992)	A	3.32	3.00	0.97	26	13	211	9.	3,58	4.00	0.52	39	59	199
8. Discussion with the patient	D	3,31	3.00	1.06	24	16	211	3.	3.68	4.00	0.50	29	69	204
9. Organisation’s explicitly stated values or ethical principles	I	3.29	3.00	1.02	33	11	209	24.	3.34	3.00	0.70	41	46	185
10. Work shift planning	W	3.29	3.00	1.19	32	16	205	20.	3.46	4.00	0.63	40	53	181
11. Changes in operational practices	W	3.28	3.00	0.70	34	2	214	8.	3.60	4.00	0.51	38	61	205
12. Negotiating with all parties involved in the problem	D	3.26	3.00	1.02	27	13	209	2.	3.71	4.00	0.49	26	72	202
13. Work unit-specific explicitly stated values or ethical principles	I	3.25	3.00	1.17	33	14	206	18.	3.47	4.00	0.61	41	53	179
14. Debriefing problematic situations in a working group	D	3.17	3.00	1.08	24	14	210	7.	3.61	4.00	0.57	30	65	194
15. Rearrangement of work duties within a unit	W	3.15	3.00	0.83	31	3	214	15.	3.51	4.00	0.54	45	53	199
16. Discussing with the patient’s family members	D	3.14	3.00	1.09	27	12	205	5.	3.62	4.00	0.54	32	65	191
17. Health Care Act (1326/2010)	A	3.10	3.00	1.02	25	9	208	16.	3.49	4.00	0.57	43	53	181
18. Discussing with other health care professionals	D	3.08	3.00	0.86	24	5	212	21.	3.42	3.00	0.58	49	46	200
19. Professional codes of ethics	I	2.88	3.00	1.04	16	9	210	26.	3.31	3.00	0.66	48	42	181
20. Work rotation	W	2.86	3.00	0.96	23	3	213	10.	3.56	4.00	0.56	37	59	187
21. Personal Data Act (523/1999)	A	2.79	3.00	1.09	16	8	207	19.	3.47	4.00	0.58	44	51	177
22. Use of extra personnel	W	2.76	3.00	0.99	22	2	211	23.	3.38	3.00	0.68	41	49	180
23. Some other act	A	2.65	3.00	1.38	21	11	113	14.	3.53	4.00	0.57	40	56	80
24. Rearrangement of work duties between units	W	2.65	3.00	0.94	19	0	211	22.	3.38	3.00	0.56	54	42	178
25. Occupational health and safety organisation	O	2.62	3.00	0.90	12	2	214	35.	3.03	3.00	0.73	52	26	183
26. Professional guidance	O	2.57	3.00	1.08	18	3	211	25.	3.33	3.00	0.66	47	43	184
27. Ethics literature, articles	I	2.56	3.00	0.91	13	1	210	30.	3.21	3.00	0.63	58	32	170
28. Occupational health care	O	2.55	3.00	0.95	14	1	214	36.	3.03	3.00	0.78	47	29	180
29. The ETENE ethical recommendations for health care	I	2.38	2.00	1.02	12	1	205	28.	3.26	3.00	0.64	54	36	145
30. Participation in training concerning ethics	O	2.36	2.00	0.81	8	0	210	29.	3.23	3.00	0.65	56	34	173
31. Code of ethics for nurse managers	I	2.34	2.00	1.04	12	2	199	31.	3.16	3.00	0.69	52	33	135
32. Regulation of patient flow	W	2.32	2.00	1.12	9	5	199	32.	3.09	3.00	0.79	51	31	147
33. Transferring a person to another unit	W	2.27	2.00	0.82	4	0	211	27.	3.31	3.00	0.65	50	41	171
34. Mental Health Act (1116/1990)	A	2.26	2.00	1.21	9	7	202	17.	3.48	4.00	0.65	40	55	128
35. Ethics-related introductions in meetings	D	1.99	2.00	0.92	3	1	208	33.	3.05	3.00	0.67	60	23	120
36. Theologian or other specialist in spiritual matters	O	1.78	2.00	0.90	4	0	210	38.	2.93	3.00	0.80	57	21	126
37. Ethics checklists	I	1.60	1.00	0.86	3	1	196	34.	3.05	3.00	0.76	54	27	84
38. Ethics specialist	O	1.24	1.00	0.56	1	0	200	37.	2.97	3.00	0.77	55	23	65
39. Ethics committee dealing with nursing solutions (excluding research ethics)	O	1.23	1.00	0.51	2	0	200	39.	2.88	3.00	0.81	47	23	66
40. Keeping an ethics journal	D	1.16	1.00	0.40	0	0	207	40.	2.49	2.00	0.93	34	15	74

categories: *D* Discussions and deliberation, *W* Work arrangements, *A* Acts and degrees, *I* Written instructions and ethical principles, *O* Outside experts

^a^Higher values mean more often

^b^ Higher values mean more useful

### Most useful methods of solving ethical problems

The NMs who answered the question on usefulness considered most of the methods to be somewhat or very useful. However, for nine out of 40 methods the answer was empty in at least one third of the responses. The mean scores of usefulness were calculated both for the sum variables and the individual methods.

Among the sum variables, the mean score for discussions and deliberation was the highest, while acts and degrees were considered almost equally useful. There were statistically significant differences in usefulness between all other sum scores except discussions and deliberations vs. acts and degrees, as well as acts and degrees vs. work arrangements (Table 1).

As for the single methods, discussion with nurses was considered the most useful method; all participants considered it somewhat or very useful. Negotiating with all parties involved in the problem, discussion with the patient and discussion with manager colleagues were considered somewhat or very useful by 98% of the NMs. Altogether 15 methods had a mean value above 3.5, the scale being 1–4 (Table 2).

### Background factors associated with the frequency of using the methods

Multivariate regression analysis was used to find out associations between work-related or socio-demographic background factors or the frequency of ethical problems and the frequency of use or considered usefulness of the methods for solving ethical problems.

Socio-demographic background factors were associated with the frequency of using methods in three dimensions. First, NMs who had participated in continuing education concerning ethics over the last 2 years used more often outside experts, written instructions and ethical principles. Secondly, written instructions and ethical principles were more often used by NMs who had higher education, in other words, a university degree. Finally, NMs in ward management used acts and decrees as well as work arrangements less often than NMs in middle or strategic management (Table 3).

**Table 3:** Multivariable regression models of the frequency of use of methods in solving ethical problems

Independent variables		Dependent variables				
		Methods used in solving ethical problem ^a^				
		Discussion and deliberation	Use of outside experts	Written instructions and ethical principles	Acts and degrees	Work arrangements
		beta (SE)	beta (SE)	beta (SE)	beta (SE)	beta (SE)
Participating in education concerning ethics in last 2 years						
Yes		–	0.17 (0.08)	0.35 (0.11)	–	–
No ^b^		–	0	0	–	–
p ^c^		–	0.0269	0.0012	–	–
Education						
University of applied sciences or other institute		–	–	−0.25 (0.10)	–	–
University ^b^		–	–	0	–	–
p ^c^		–	–	0.0115	–	–
Position in organisation						
Ward management		–	–	–	− 0.36 (0.13)	− 0.20 (0.09)
Middle or strategic management ^b^		–	–	–	0	0
p ^c^		–	–	–	0.0082	0.0257
Sum score of work-related background factors		–	–	0.23 (0.10)	–	–
p ^c^		–	–	0.0254	–	–
Frequency of ethical problems related to patients ^d^		0.16 (0.07)	–	–	–	–
p ^c^		0.021	–	–	–	–
Frequency of ethical problems related to nursing staff^d^		–	–	–	–	0.24 (0.10)
p ^c^		–	–	–	–	0.0198
Difficulty of ethical problems related to nursing staff ^e^		− 0.17 (0.06)	–	− 0.25 (0.09)	− 0.28 (0.10)	–
p ^c^		0.0064	–	0.0046	0.0068	–
Frequency of ethical problems related to organisation ^d^		–	0.15 (0.06)	0.28 (0.09)	0.46 (0.10)	0.22 (0.08)
p ^c^		–	0.009	0.0017	<.0001	0.0047
Difficulty of ethical problems related NM her/himself ^e^		–	− 0.09 (0.04)	–	–	− 0.16 (0.08)
p ^c^		–	0.0218	–	–	0.0444
100 x R^2^ (%)		5.1	8.1	18.5	16.3	12.4
p ^f^		0.0042	0.0007	<.0001	<.0001	<.0001

Empty cell (−): No significant association.

*SE* Standard error.

^a^frequency 1 = never, 5 = always

^b^reference category

^c^significance of the independent variable

^d^frequency 1 = never, 5 = daily

^e^difficulty, 1 = very easy, 5 = very difficult

^f^significance of the multivariate regression model

The sum score of work-related background factors was associated with the frequency of use of written instructions and ethical principles. The more positive the NMs’ assessment of the work-related issues, the more often the NMs used them (Table 3).

Associations were also found between the sum scores of the frequency of ethical problems encountered by NMs and the frequency of using methods. The more often NMs encountered patient-related ethical problems, the more often they used discussions or deliberations. The frequency of organisation-related ethical problems associated with the use of outside experts, written instructions and ethical principles, acts and degrees and work arrangements. Work arrangements were also used more often by NMs who encountered more ethical problems related to nursing staff (Table 3).

The sum scores of difficulty of ethical problems encountered by NMs associated with the frequency of using methods in two groups. The more difficult the NMs considered the ethical problems related to nursing staff, the less they used discussions and deliberation, written instructions and ethical principles as well as acts and degrees as problem-solving methods. The association was reverse also with difficulty of ethical problems related to the NMs themselves and using outside experts or work arrangements (Table 3).

### Background factors in association with the usefulness of the methods

Some associations were also found between socio-demographic background factors and the usefulness of the methods. NMs who had participated in continuing education concerning ethics in the last two years considered discussion and deliberation more useful than others. Older NMs considered many methods more useful than did younger ones: discussions and deliberation, written instructions and ethical principles, acts and degrees, and work arrangements (Table 4.)

**Table 4:** Multivariable regression models of the usefulness of methods in solving ethical problems

Independent variables	Dependent variables				
	Methods used in solving ethical problem ^a^				
	Discussion and deliberation	Use of outside experts	Written instructions and ethical principles	Acts and degrees	Work arrangements
	beta (SE)	beta (SE)	beta (SE)	beta (SE)	beta (SE)
Participating in education concerning ethics in last 2 years					
Yes	0.12 (0.05)	–	–	–	–
No ^b^	0	–	–	–	–
p ^c^	0.0088	–	–	–	–
Age	0.007 (0.003)	–	0.008 (0.004)	0.01 (0.004)	0.01 (0.004)
p ^c^	0.0167	–	0.0430	0.0117	0.0053
Sum score of work-related background factors	0.09 (0.04)	0.16 (0.07)	–	–	–
p ^c^	0.0410	0.0187	–	–	–
Frequency of ethical problems related to patients ^d^	–	–	–	–	−0.15 (0.07)
p ^c^	–	–	–	–	0.0450
Frequency of ethical problems related to nursing staff^4)^	0.14 (0.05)	–	–	0.35 (0.08)	0.24 (0.09)
p ^c^	0.0131	–	–	<.0001	0.0085
Frequency of ethical problems related to organisation ^d^	–	–	–	–	−0.14 (0.06)
p ^c^	–	–	–	–	0.0298
Difficulty of ethical problems related to organisation ^e^	–	–	–	0.10 (0.05)	0.13 (0.04)
p ^c^	–	–	–	0.0490	0.0029
Frequency of ethical problems related to other professional groups ^d^	–	–	–	–	0.24 (0.08)
p ^c^	–	–	–	–	0.0027
Difficulty of ethical problems related to other professional groups ^e^	0.09 (0.03)	–	–	–	
p^c^	0.0040	–	–	–	
Frequency of ethical problems related NM her/himself ^d^	−0.15 (0.04)	–	−0.18 (0.06)	−0.23 (0.07)	− 0.14 (0.06)
p ^c^	0.0010	–	0.0022	0.0006	0.0234
100 x R^2^ (%)	19.1	2.6	7.2	12.9	13.9
p ^f^	<.0001	0.0187	0.0005	<.0001	<.0001

Empty cell (−): No significant association.

*SE* Standard error.

^a^usefulness 1 = useless, 4 = very useful

^b^reference category

^c^significance of the independent variable

^d^frequency 1 = never, 5 = daily

^e^difficulty, 1 = very easy, 5 = very difficult

^f^significance of the multivariate regression model

The sum score of work-related background factors was associated with the usefulness of the methods so that the more positively the NMs assessed their work-related issues, the more useful they considered discussion and deliberation and the use of outside experts (Table 4).

The more frequently the NMs encountered ethical problems related to patients or organisation, the less useful they considered work arrangements. Instead, the more frequently the NMs encountered ethical problems related to nursing staff and other professional groups, the more useful they considered work arrangements. The frequency of ethical problems related to nursing staff was also associated with the usefulness of discussion and deliberation as well as acts and degrees. The more often the NMs encountered ethical problems related to themselves, the less useful they considered all methods except the use of outside experts (Table 4).

Some associations were also found with the degree of difficulty of the ethical problems, so that the more difficult the NMs considered the ethical problems related to organisation, the more useful they found acts and degrees and work arrangements. Additionally, the more difficult the NMs considered the ethical problems related to other professional groups, the more useful they considered discussion and deliberation (Table 4).

## Discussion

This study provided new knowledge about the solving methods of ethical problems in nursing management. The study revealed that NMs use a variety of methods when solving ethical problems, and most of methods are considered to be useful. More details of the methods and new knowledge of their usefulness were provided, which is applicable in nursing management globally. The methods that NMs use most frequently are the same as reported in the 1990s. Borawski (1994) found the most frequently used resources in ethical decision-making to be 1) nurse colleagues, 2) administrative colleagues and 3) personal values. [10]. In this study, the respondents were given 40 different resources to choose from, and the top three were the same, in different order. This result points out the quite rigid ways of solving ethical problems in nursing management.

Situation-specific, pre-defined instructions and situation or organisation-specific instructions or principles were among the ten most frequently used methods in our study. This result should encourage organisations to develop instructions for ethically difficult situations. Instead, the code of ethics for nurse managers was not used very often, and it seems that its use is not well established. Nurse managers’ own codes still seem to be quite a rare and new phenomenon, so there does not appear to be earlier research about their use. Professional codes of ethics were used more often, but they were not among the most often used methods, either. These results are similar to the results of Cooper et al. [9] and Borawski [10].

Ethics committees dealing with nursing solutions (excluding research ethics) were seldom used; 80% of participants had never used them. In addition, 81% had never used ethics specialists to solve an ethical problem. There are probably two explanations: these methods were either not available or were not used, as has been found to be the case among nurses and physicians [22, 23]. In our study, the participants were not asked if they had a possibility to use ethical committees or ethical specialists in their organisations. It is possible that even if ethical committees or specialists are available, NMs may not be attuned to using them to solve ethical issues concerning personnel management or organisational issues. In some studies, about 20% of NMs have mentioned the use of ethics committees [1, 11], which seems to be more than in our study, but those studies only asked about use, not its frequency.

NMs consider most of the methods used to solve ethical problems useful. There were many methods that were considered to be very useful but were seldom used. Some of them were probably useful in situations which are uncommon, or the methods were not easily available.

There are various associations between the ethical problems encountered and the methods used for solving them. For example, discussion and deliberation are used the more frequently the more NMs encounter patient-related problems. It was not confirmed here that discussions are used most with patient-related problems, but this gives a strong indication of that.

NMs should have versatile knowledge of methods to deal with ethical problems in their own work, but also to be able to support and advise their subordinates in solving ethical problems. When nurses need consultation on an ethical problem, nurse manager is one of the most often used resources [13]. The results indicate that NMs with higher education, higher position or recent ethics education use more often other methods than discussions. The use of outside experts, written instructions and ethics literature should be relevant options in ethical problem-solving, and education is needed to increase their use. Although discussions are important and useful, experts of ethics and theoretical information expand the basis of ethical decision-making.

Some limitations in this study are worthy of attention. First, the EProNuMa questionnaire was developed for this study so there is no previous information about its reliability and validity. The development followed the process including item generation, based on the literature and interviews [2], and content validity testing by using expert analysis of 5 nurse managers at different levels and with different work experience. Furthermore, the EProNuMa was also pilot tested by 15 nurse managers. Internal consistencies of the sum variables of the frequency and the difficulty of ethical problems were at good level (Table 1) [24]. Secondly, the response rate was quite low (20%) but adequate for Webropol surveys in management [25]. However, the sample was representative: a large group of NMs at different levels of management and different kinds of healthcare organisations nationwide. The sample corresponds closely with the overall group of nurse managers in Finland in terms of mean age (50.6 years) and gender (female 94%) [26].

## Conclusions

Ethical problems will always exist in healthcare, but in the rapidly changing and complex present-day healthcare context there is an increasing need for methods to solve them. Diverse useful methods should therefore exist and be accessible for improving ethical problem solving. Ethical problems are associated with adverse outcomes [4], and healthcare organisations would therefore benefit from a decrease in the number of ethical problems.

Discussions and negotiations are found to be useful methods when solving ethical problems. Moral case deliberation (MCD) is quite a new and more organised way of discussing ethical issues. Discussions in general, including MCD, are thus worthy of more studies to explore what kind issues are discussed and what kind of reasoning is used.

Situation-specific instructions and unit- or organisation-level ethical guidelines seem to be commonly used methods and they should be produced to meet the specific needs of different work units. The code of ethics of nurse managers should be developed and publicised so that NMs would have ethical guidelines allocated to their work.

Nursing managers may benefit from the possibility to consult ethical experts or committees when encountering ethical problems. The experts and committees can provide an outside perspective to ethical issues, and they could also collect data on handling ethical problems to help future problem-solving in similar cases. Systematically gathered data should also be utilised in research to find out what kind of methods are used to solve different kinds of problems.

## Data Availability

The datasets used and/or analyzed during the current study are available from the corresponding author on reasonable request.

## Abbreviations

MCD: Moral Case Deliberation
NM: Nurse Manager
